# Cure of Spina Bifida by Application of Collodion

**Published:** 1860-01

**Authors:** 


*Cure of Spina Bifida by application of Collodion.*

In a late number we alluded to Dr. Brainard's success in the treatment of spina bifida by iodine injections. A case which was cured by the application of collodion is reported in the *Journal fur Kinderhrankheiten.* The child was seven weeks old, and the tumor was of the size of a small orange. When the fluid was pressed into the spine, the patient had pain, and the muscles of the face were convulsed. The tumor was covered with collodion, at first mixed with an equal quantity of castor oil, afterwards with a mixture of three parts of collodion and one of oil, and finally with pure collodion. The tumor disappeared at the end of three weeks. The patient was treated by Dr. Behrend. "It should be added," says the reporter, "that the child having presented cerebral symptoms, was also treated with calomel, to which, perhaps, a share of the cure is due." We should be inclined to doubt this assertion. In our opinion the calomel was at least nugatory, if not injurious to the pro gress of recovery.—*Boston Medical and Surgical Journal.*

